# Structure and Lateral Organization of Phosphatidylinositol 4,5-bisphosphate

**DOI:** 10.3390/molecules25173885

**Published:** 2020-08-26

**Authors:** Luís Borges-Araújo, Fabio Fernandes

**Affiliations:** 1 iBB—Institute for Bioengineering and Biosciences, Instituto Superior Técnico, Universidade de Lisboa, 1049-001 Lisbon, Portugal; fernandesf@tecnico.ulisboa.pt; 2 Department of Bioengineering, Instituto Superior Técnico, Universidade de Lisboa, 1049-001 Lisbon, Portugal

**Keywords:** phosphoinositides, lipid domains, calcium-induced clustering

## Abstract

Phosphatidylinositol 4,5-bisphosphate (PI(4,5)P_2_) is a minor but ubiquitous component of the inner leaflet of the plasma membrane of eukaryotic cells. However, due to its particular complex biophysical properties, it stands out from its neighboring lipids as one of the most important regulators of membrane-associated signaling events. Despite its very low steady-state concentration, PI(4,5)P_2_ is able to engage in a multitude of simultaneous cellular functions that are temporally and spatially regulated through the presence of localized transient pools of PI(4,5)P_2_ in the membrane. These pools are crucial for the recruitment, activation, and organization of signaling proteins and consequent regulation of downstream signaling. The present review showcases some of the most important PI(4,5)P2 molecular and biophysical properties as well as their impact on its membrane dynamics, lateral organization, and interactions with other biochemical partners.

## 1. Introduction

**Figure 1:**
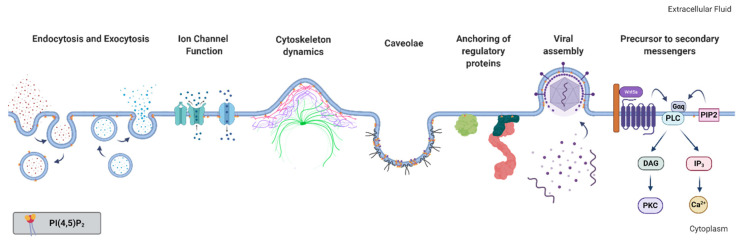
Membrane processes associated with or dependent on phosphatidylinositol 4,5-bisphosphate (PI(4,5)P_2_). Figure created with BioRender.com.

The plasma membrane is a complex structure tasked with enclosing the cell and separating it from the surrounding environment. While biomembranes provide structure and define the boundaries of the cell, their dynamic biochemical and biophysical characteristics also allow them to regulate traffic and communication to and from the cytosol, organize reaction sequences, and promote cellular processes. These biophysical properties are defined not only by the intrinsic physical and chemical properties of the lipids, proteins, and other components but also by their complex set of interactions. This complexity holds the key to many key cellular processes. A lipid that stands out in the landscape of the eukaryotic plasma membrane is phosphatidylinositol 4,5-bisphosphate (PI(4,5)P_2_). PI(4,5)P_2_ is the most abundant phosphoinositide in mammalian cells and is found primarily in the inner leaflet of the plasma membrane. It has also been found in endosomes, in the endoplasmic reticulum, and in the nucleus [1]. While its role as a source of secondary messengers during signaling events is known for decades [2], evidence has accumulated through time of its importance as an intact phospholipid for defining plasma membrane identity in eukaryotic cells. Due to its very large headgroup and multivalent negative charge, PI(4,5)P_2_ acts almost like an electrostatic beacon, interacting specifically or non-specifically with several molecular entities such as membrane proteins, other lipids, cellular cations, etc. As a result of its particular biophysical properties, it is a major regulator of a wide spectrum of plasma membrane events, including cell adhesion and motility [3,4], ion channel transport [5,6], vesicle endocytosis [7,8,9,10,11], and exocytosis [8,12,13,14] (Figure 1). 

This wide-ranging reach of PI(4,5)P_2_ as a critical functional lipid has made it an important research focus over the last decades in cell biology and more specifically in areas such as neuroscience, virology, and biophysics. In this review, we will focus on the physical, chemical, and structural properties of PI(4,5)P_2_. The impact of these properties on membrane dynamics and interactions with PI(4,5)P_2_ biochemical partners will also be described. We will give particular focus to PI(4,5)P_2_ conformation dynamics, lateral organization, and the monodisperse PI(4,5)P_2_–PI(4,5)P_2_ nanodomain duality. As mentioned above, PI(4,5)P_2_ is also found in the nuclear membrane, where it is thought to contribute to compartmentalization [15,16], as well as on membraneless organelles within the nucleus [15,16]. However, this review will focus on the behavior and properties of PI(4,5)P_2_ in the plasma membrane (PM) and PM mimicking membranes.

## 2. The Phosphoinositide Family

PI(4,5)P_2_ is a member of the phosphoinositide (PI) lipid family. PIs are a small group of glycerophospholipids derived from phosphatidylinositol. These lipids consist of a characteristic inositol headgroup, which can undergo reversible phosphorylation and dephosphorylation, leading to the formation of seven distinct phosphorylated species. While the parent lipid phosphatidylinositol represents roughly 10% of total membrane phospholipids in the eukaryotic cell, the phosphorylated derivatives account only for around 2–3% [1], with PI(4)P and PI(4,5)P_2_ representing the bulk of these lipids [17]. Each of these seven species has a distinct subcellular distribution with predominant localization in subsets of membranes. Additionally, within a given membrane the localization of a specific PI can be heterogeneous. Many PIs are overall in low abundance in the membrane but they can be found at high local concentrations in membrane domains not readily detected by conventional techniques [3,17]. For a historical review on inositol lipids, see Irvine (2016) [18]. Over the last couple of decades, PIs have been found to be one of the most ubiquitous signaling entities in eukaryotic metabolism. Their reach extends from controlling organelle biology to regulating cellular growth. Due to this all-encompassing reach, they have also been linked to a number of human diseases. In fact, the inositide signaling pathway is considered a promising pharmaceutical target. For an excellent review on the major developments on PI cellular biology and their impact on disease, see Balla (2013) [3]. 

## 3. PI(4,5)P_2_ Structure

### 3.1. Headgroup Conformation

**Figure 2:**
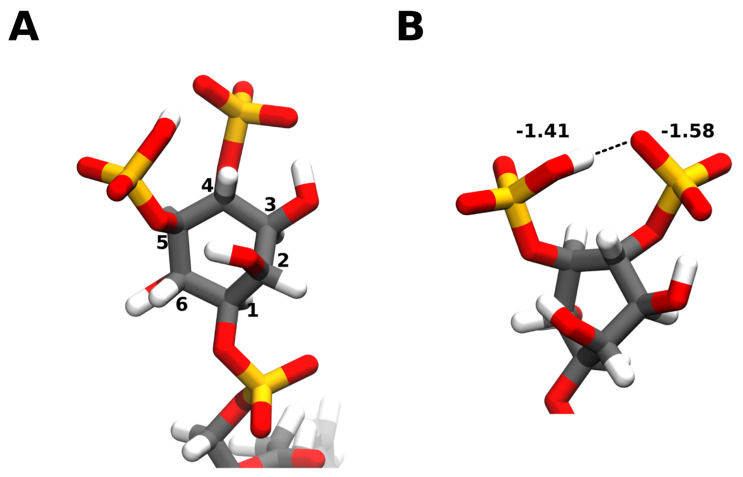
PI(4,5)P2 headgroup features. The PI(4,5)P_2_ headgroup consists of *myo*-inositol ring where every hydroxyl substituent is at the equatorial position except for the hydroxyl in the position 2 of the ring, which is in an axial position. In the case of PIP2, the hydroxyls in positions 4 and 5 are enzymatically phosphorylated. It is linked to the diacylglycerol (DAG) backbone via a phosphodiester bond in position 1 (**A**). At pH 7.0, one of the phosphodiester proton dissociates, and the one remaining is shared between the two vicinal phosphomonoester groups. In terms of potential charge this means that, at pH 7.0, the charges would be −1.58 and −1.41 for the phosphomonoester groups at positions 4 and 5, respectively [19]. The lower charge of the 5-phosphomonoester group is attributed to a network of intramolecular hydrogen bonds that it is engaged in, which stabilize the proton (**B**). Carbon atoms are colored in grey, hydrogen in white, oxygen in red, and phosphorus in orange. Snapshots obtained from a simulation of a bilayer consisting of 95:5 mol ratio 1-palmitoyl-2-oleoyl-*sn*-glycero-3-phosphocholine (POPC): PI(4,5)P2, using the CHARMM36m forcefield run in GROMACS2019. Images were modeled using VMD.

The core PI(4,5)P_2_ structure descends from its “parent” lipid, phosphatidylinositol. At the core, it consists of a *myo*-inositol headgroup. There are 9 existing isomers of inositol but the *myo*-inositol isomer is the most commonly found in eukaryotic cells. In its most stable conformation, it assumes the chair conformation where every hydroxyl substituent is at the equatorial position except for the hydroxyl in the position 2 of the ring, which is in an axial position. The *myo*-inositol moiety is typically linked to the diacylglycerol (DAG) backbone via a phosphodiester bond in position 1. This leaves the hydroxyl groups in positions 2 to 6 open. However, only positions 3, 4, and 5 can be enzymatically phosphorylated to yield the 7 phosphorylated PI species. PI(4,5)P_2_ is the result of phosphorylation in positions 4 and 5 of the headgroup by specific kinases and phosphatases (Figure 2A). 

In mammals, the majority of PI(4,5)P_2_ in the plasma membrane is synthesized from PI(4)P by type I phosphatidylinositol-4-phosphate 5-kinases (PIP5Ks) (α, β, and γ) [20]. Type II phosphatidylinositol 5-phosphate 4-kinase (PIP4K) (α, β, and γ) phosphorylate PI(5)P_2_ to synthesize a quantitatively minor pool of PI(4,5)P_2_ localized in the Golgi [1,21,22]. It can also be produced by the dephosphorylation of PI(3,4,5)P_3_ catalyzed by phosphatase and tensin homolog protein (PTEN) and phosphatidylinositol 3,4,5-trisphosphate 3-phosphatase (TPIP) (α, β, and γ) [23,24]. PI(4,5)P_2_ hydrolysis is controlled by specific 4’- or 5’-phosphatases or by phospholipase breakdown in response to various stimuli. Dephosphorylation by specific phosphatases (primarily 5’-phosphatases) controls PI(4,5)P_2_ steady-state levels and controls the extent of its signaling. Additionally, cleavage by phospholipases, such as phospholipase C (PLC), control PI(4,5)P_2_ levels and originate metabolites that propagate and amplify cellular signaling. PI(4,5)P_2_ levels, in general, are the result of a complex interplay of many cellular enzymes. While PI(4,5)P_2_ metabolism falls out of the scope of this review, more information can be found elsewhere [3,25]. 

### 3.2. Membrane Conformation Dynamics

In terms of molecular structure when inserted into the membrane, there are surprisingly very few experimental studies probing PI(4,5)P_2_ dynamics. Since the dynamics of phosphatidylinositol or of the mono-phosphorylated PI(4)P has been addressed to some extent, we can estimate some of PI(4,5)P_2_ properties from the behavior of these closely related precursors. At the insertion depth of phosphatidylinositol and PI(4)P, when inserted into the membrane and in the absence of any interactions with other chemical partners, it is believed that the phosphodiester bond is located roughly at the same depth as the phosphodiester of phosphatidylcholine. Additionally, evidence points out that the phosphodiester bond remains roughly parallel when compared to the membrane normal [26,27,28,29].

PI(4,5)P_2_ headgroup tilt seems to be significantly impacted by phosphorylation. For the case of phosphatidylinositol, the headgroup is roughly perpendicular to the membrane plane, with the C4 hydroxyl as the most exposed to the water layer despite a slight tilt being observed arising from an intramolecular hydrogen bond established between the C2 hydroxyl and the pro-R-oxygen of the phosphodiester phosphate [26,27,28,30]. This is the result of the glyceryl-phosphate-inositol link being always very close to a trans, trans, trans, gauche-conformation, which brings the two hydrogen bond partners together. Interestingly, the formation of this hydrogen bond appears to be crucial for the recognition by PLC, however, it is not yet clear if it is formed when phosphatidylinositol is aggregated [27]. In the case of PI(4)P, the headgroup tilt is more significant [26,28,29], and authors suggest it might be also due to the establishment of electrostatic interactions between the negatively charged phosphate and the positively charged choline headgroups from the neighboring lipids. For PI(4)P, due to this more significant tilt, the C5 hydroxyl is the most accessible to the water layer. 

**Figure 3:**
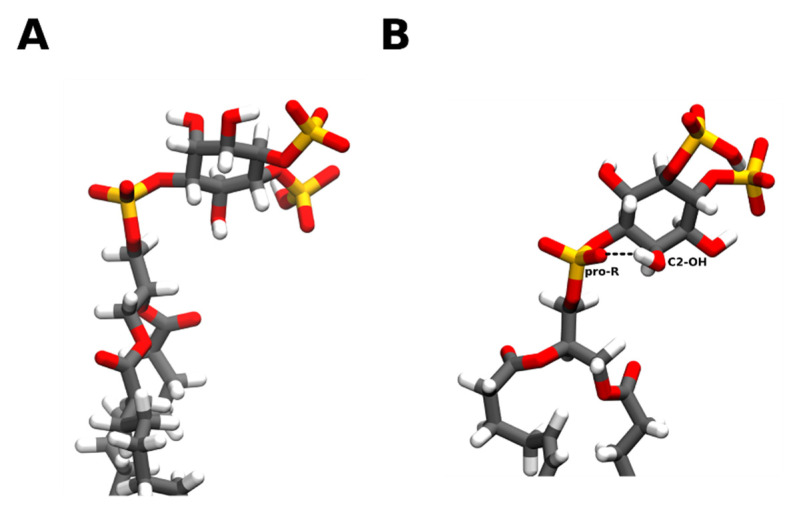
Examples of PI(4,5)P_2_ headgroup tilt when inserted into a phospholipid membrane. PI(4,5)P_2_ presents a significant headgroup tilt, when inserted into a bilayer, ranging from almost parallel to the membrane plane (**A**) to a more conservative ~40° tilt (**B**). Whilst the more dramatic headgroup tilt appears to be favored from interactions between its negatively charged phosphate groups and the positively charged membrane surface, the more moderate tilt surges from the establishment of intramolecular hydrogen bonds between the C2 hydroxyl and the pro-R-oxygen of the phosphodiester phosphate. Carbon atoms are colored in grey, hydrogen in white, oxygen in red, and phosphorus in orange. Snapshots obtained from a simulation of a bilayer consisting of 95:5 mol ratio POPC: PI(4,5)P_2_, using the CHARMM36m forcefield run in GROMACS2019. Images were modeled using VMD.

In a Variable Angle Sample Spinning NMR study [29], PI(4,5)P_2_ membrane conformation was studied in a membrane-like environment consisting of neutral alkyl-poly(ethylene)glycol and long-chain alcohols. All possible conformations obtained showed a much more pronounced headgroup tilt for PI(4,5)P_2_ than for PI(4)P, where the PI(4,5)P_2_ headgroup would be laying almost parallel to the membrane surface. As this cannot arise from specific electrostatic interactions in this membrane model, it is likely that this is the result of more subtle effects such as water- or alcohol-mediated hydrogen bonding. As the analysis of NMR measurements of complex systems (such as PIs) is error-prone and the “membrane matrix” used is far from being biological relevant, the authors of this study could not be definitive in their conclusions regarding PI(4,5)P_2_ orientation. Nevertheless, they were able to replicate what had been previously observed for PI(4)P in other membrane mimetics. If these observations are replicated in more relevant conditions, they will challenge the more established “concept” of how PI(4,5)P_2_ is structurally displayed in the membrane and how it interacts with protein partners. All-atom molecular dynamics simulations of PI(4,5)P_2_ in lipid membranes show a well-defined average head-tail angle of roughly 100°, indicating that the headgroup would lie mostly flat along the membrane in agreement with the previous studies [31] (Figure 3). Poisson Boltzmann MD simulations, however, show a more conservative tilt of roughly 40°. 

Overall, there are strong hints that, in the absence of interactions with other biochemical partners, PI(4,5)P_2_ when inserted into a membrane has its headgroup lying tilted over the membrane, however, the extent of this tilt is still yet to be fully understood and likely depends not only on PI(4,5)P_2_ intrinsic properties but also on the interactions with the neighboring lipid molecules.

### 3.3. Headgroup Charge

An important aspect that is closely related to the conformation of PI(4,5)P_2_ is the charge state of each of its headgroup phosphate groups. Whilst the charge state of the headgroup linking phosphate group is well defined at physiological pH (pKa between 1 to 3) [32], the headgroup phosphate charges are much more volatile. The charge state of these groups has mostly been studied experimentally for PI(4,5)P_2_ in micelles and small unilamellar vesicles (SUVs), via 31P–NMR and the dependency of the chemical shift on the pH. Typically, PI(4,5)P_2_ is considered to have approximately four negative charges at cytosolic pH. This result was inferred from the determination of the pKa of each headgroup phosphate in either pure or mixed vesicles of PI(4,5)P_2_ using 31P–NMR [33]. These experiments determined a pKa value for the first protonation of roughly 6.7 and 7.7 for the phosphomonoester groups at position 4 and 5, respectively. In terms of potential charge, this would mean that at pH 7.2, the 5-phosphomonoester would be partially protonated at charge −1, the 4-phosphomonoester would be fully deprotonated with charge −2, and the phosphodiester would have charge −1. 

However, studies have since shown that the PI(4,5)P_2_ headgroup ionization behavior (as well as for other phosphoinositides) cannot be accurately described by a Henderson-Hasselbalch mechanism [19]. In the more detailed mechanism that was proposed by the authors [19], at pH values close to 4–5, both phosphomonoester groups are mono-protonated as previously described. Upon increasing the pH, one proton dissociates, whilst the remaining one is shared between the two vicinal phosphomonoester groups (Figure 2B). In terms of potential charge this means that, at pH 7.0, the charges would be −1.58 and −1.41 for the phosphomonoester groups at positions 4 and 5, respectively. The lower charge of the 5-phosphomonoester group is attributed to the fact that it establishes a hydrogen bond interaction with the hydroxyl group in the position 6, which in turn, is also forming a long-range hydrogen bond with the phosphodiester group in the position 1. This weakens the first hydrogen bond slightly and thus the proton binds to the 5-phosphomonoester more tightly. These results gave a much more detailed look at the charge distribution of PI(4,5)P_2_ and at the complex network of intra- and intermolecular hydrogen bonds that lead to the dissipation of the headgroup charge and are, very likely, part of the reason for why repulsion between phosphoinositides is much weaker than expected. 

A fact that is often overlooked is how the interaction of PI(4,5)P_2_ with neighboring molecules influences its charge distribution. In vivo, PI(4,5)P_2_ is constantly in interaction not only with its neighboring lipids but also with proteins and cationic ions. The complex network of interactions formed by PI(4,5)P_2_ with these partners leads to a greater distribution of its charge, effectively altering its headgroup charge. A study has shown that, in the absence of divalent cations, lipids with hydrogen bond donor capabilities could influence PI(4,5)P_2_ headgroup charge [34]. Phosphatidylethanolamine (PE) was shown to influence the first step of deprotonation of the PI(4,5)P_2_ headgroup, most likely by interacting preferably with the 5-phosphate. In the presence of PI, the first step of deprotonation was not affected, however, a lower degree of ionization was observed for both phosphomonoester groups for the removal of the last shared proton. Curiously, phosphatidylserine (PS) was not shown to affect PI(4,5)P_2_ headgroup ionization significantly. This study clearly showcases how the PI(4,5)P_2_ local environment can affect PI(4,5)P_2_ headgroup charge, a crucial feature responsible for a lot of its biological function.

### 3.4. Acyl-Chain Composition

The acyl-chain composition of lipids often plays an important functional role. These roles can be defined by specific interactions with proteins or by simply changing the overall biophysical properties of the surrounding membrane. In general, fatty acid profiles vary between phospholipid classes, tissues, and species and can also vary over time with dietary habits, stimuli, or disease. Like many other lipids, PI(4,5)P_2_ is also subject to these effects. In mammals, the phosphorylated *myo*-inositol headgroup is typically bound to a DAG moiety, which consists of two fatty acid chains bonded to a glycerol molecule via ester bonds at positions *sn*1 and *sn*2. The major fatty acid profile observed for PI(4,5)P_2_ in mammals consists of 1-stearoyl-2-arachidonyl (18:0/20:4) [23]. Curiously, inositol-phosphate headgroups coupled to ceramide have also been identified in fungi, plants, and protozoa [35], however, we will focus only on the mammal relevant DAG-bound species in this review. 

The 18:0/20:4 acyl-chain profile consists of up to 70% of the total PI(4,5)P_2_ lipid pool in some cell lines, especially in brain tissue. This enrichment is likely the combined outcome of substrate specificity for 1-stearoyl-2-arachidonyl-glycerol in several enzymes in the phosphatidylinositol cycle and the remodeling of phosphoinositide acyl-chains via the Land’s cycle [36]. A more detailed look at how the cell might maintain this enrichment can be seen in this review by D’Souza et al. (2014) [36]. However, PI(4,5)P_2_ still has a broad distribution profile ranging from highly unsaturated chains to fully saturated ones [37]. These less abundant species become more prevalent in response to certain stimuli [38], stress [37,38], aging [37], or in cancer [39]. In these cases, fully saturated and mono-unsaturated compositions increase significantly in concentration, in some cases even surpassing the canonical 1-stearoyl-2-arachidonyl (18:0/20:4) composition [38].

However, why do cells spend so many resources in maintaining this particular acyl-chain composition? And why does the acyl-chain profile shift, sometimes dramatically, in specific conditions? The biological functions behind the enrichment in 18:0/20:4 are still not very clear. The enrichment in this configuration seems to be particularly prevalent in brain cells, where PI(4,5)P_2_ has been associated with several stages of both endocytosis and exocytosis and has been considered an important mediator of synaptic vesicle trafficking [40]. It has been shown that arachidonate, and other polyunsaturated fatty acids such as docosahexaenoate (22:6), at the *sn*2 position, facilitate membrane shaping and fission activities. Additionally, asymmetric *sn*1-saturated-*sn*2-polyunsaturated phospholipids have been shown to provide efficient membrane vesiculation whilst maintaining lower membrane permeability [41]. These properties might provide significant mechanical benefits in these particular tissues. Of course, arachidonic acid in particular has biological activity of its own in addition to serving as the precursor for the synthesis of eicosanoids, such as prostaglandins and leukotrienes [42]. Overall, this particular theme has been given little attention thus far, however, it could be the key for some of PI(4,5)P_2_ multifunctionality. 

## 4. Lateral Organization of PI(4,5)P_2_

Having looked at the core structural properties of PI(4,5)P_2_ we now turn to its organization in the plasma membrane. As PI(4,5)P_2_ is engaged in a multitude of cellular functions occurring in parallel, its levels must be tightly regulated to avoid significant fluctuations of its total plasma membrane concentration. This implies that the simultaneous regulation of these cellular functions by PI(4,5)P_2_ must occur through the presence of multiple localized pools of this phospholipid in the plasma membrane [43]. PI(4,5)P_2_ lateral organization in cells has been studied through a variety of techniques from fluorescence correlation spectroscopy (FCS) and fluorescence recovery after photobleaching (FRAP) to atomic force microscopy (AFM). In FCS experiments carried out in Rat1 fibroblasts and HEK cells, researchers microinjected micelles of fluorescent labelled-PI(4,5)P_2_ into cells and showed that the diffusion coefficient of PI(4,5)P_2_ in these cells is significantly lower than expected for free phospholipids. The simplest interpretation of this result is that approximately two-thirds of PI(4,5)P_2_ in the inner leaflet of the plasma membrane is somehow sequestered [44]. Studies in PC12 cells have also shown, using Stimulated emission depletion (STED) microscopy [45] and Stochastic optical reconstruction microscopy (STORM) imaging techniques [46], that PI(4,5)P_2_ is highly enriched in nanometer-sized membrane domains, specific to this cellular model. 

In fact, while the presence of segregated PI(4,5)P_2_ pools can be partly explained by localized PI(4,5)P_2_ synthesis and degradation through several kinases and phosphatases [47], it is also evident that membrane diffusion rates, in the absence of significant obstacles for diffusion, will always be higher than concentration changes due to enzymatic activity causing PI(4,5)P_2_ to diffuse away faster than it can be produced. This means that it is unlikely that local synthesis can result in significant changes in the submicroscopic organization of PI(4,5)P_2_ in the membrane [43]. PI(4,5)P_2_ interactions with other cellular binding partners could alternatively explain the observed lateral organization of this phosphoinositide. Interactions with proteins, divalent cations, cholesterol, and the cytoskeleton are the ones most likely to have such an impact. In this review, we will give particular attention to the often neglected effect of divalent cations on the lateral organization of PI(4,5)P_2_.

### 4.1. Sequestration by Proteins

One way to explain PI(4,5)P_2_ lateral organization in the plasma membrane of cells is that proteins can act as reversible buffers, binding much of the PI(4,5)P_2_ present and then releasing it locally in response to specific signals [48]. Theoretical simulations predict that such sequestration can be achieved not only through specific interactions with PI(4,5)P_2_ but also through nonspecific electrostatic interactions. In fact, polybasic proteins are able to sequester a lipid with a valence of ~4 (such as PI(4,5)P_2_) 1000-fold more effectively than a lipid with a valence of ~1 (such as PS) [49,50]. Due to its highly negatively charged headgroup, PI(4,5)P_2_ was confirmed to interact strongly with polybasic stretches of amino acid residues [43,51]. Through these polybasic stretches, several proteins were found to laterally sequester PI(4,5)P_2_ molecules in a reversible manner [52,53]. For an efficient buffering of PI(4,5)P_2_ levels, these proteins would have to be present at a concentration comparable to PI(4,5)P_2_, localize to the plasma membrane and be able to bind PI(4,5)P_2_ with high affinity while being able to release it in response to stimuli. Proteins such as myristoylated alanine-rich C-kinase substrate (MARCKS) [50,53,54], Growth Associated Protein 43 (GAP43) [48,55], CAP23 [48], among many others, have been shown to be able to sequester PI(4,5)P_2_ in such a manner. In the case described above of PI(4,5)P_2_ domains detected in PC12 cells, these were found to be associated with the sequestration of PI(4,5)P_2_ to clusters of the SNARE protein syntaxin-1 [45,56,57]. This sequestration by syntaxin-1 is critical for the regulation of SNARE-dependent membrane fusion [58,59].

Employing fluorescence and electron paramagnetic resonance spectroscopic tools, McLaughlin, Cafiso, and co-workers [50,52] showed that a 24 aa peptide corresponding to the effector domain of MARCKS was able to efficiently sequester an average of 3 PI(4,5)P_2_ molecules through non-specific electrostatic interactions. Importantly, this sequestration occurred even in the presence of physiological concentrations of the monovalent acidic phospholipid PS, confirming theoretical predictions. MARCKS sequestration of PI(4,5)P_2_ has been shown to be important in the PI(4,5)P_2_ mediated activation of TRPC-family Ca^2+^ channels [60], in the endocytosis of the amyloid precursor protein (APP) [61], and in the synaptic clustering of PI(4,5)P_2_ [62]. 

### 4.2. PI(4,5)P_2_ Interactions with Divalent Cations

Several studies have shown that PIs and PI(4,5)P_2_, in particular, are able to establish strong electrostatic interactions between their negatively charged headgroups and divalent cations. In the cellular PI(4,5)P_2_ context, calcium and magnesium stand out. Calcium is a common player in signal transduction and a second messenger in cells. Its levels are strictly controlled and maintained at low levels in the cytosol, with normal intracellular levels at around 100 nM (20,000 fold lower than extracellular levels) [63]. Upon stimulation, however, several signal transduction pathways can lead to transient increases of intracellular calcium concentration up to around 1 μM, with local concentrations in the vicinity of open calcium channels reaching hundreds of μM, before being regulated back to normal levels [64]. In fact, PI(4,5)P_2_ has been reported to be associated with a variety of Ca^2+^ channels and a great number of these require PI(4,5)P_2_ for proper function [3]. Magnesium, on the other hand, is a less studied modulator of cell function. Magnesium levels are well buffered in a narrow millimolar range between 0.25 mM and 1 mM [65,66] and are thus kept at a much higher concentration than those of calcium. Both divalent cations have been shown to bind strongly to PI(4,5)P_2_ and influence its lateral organization dramatically as discussed below. 

**Figure 4:**
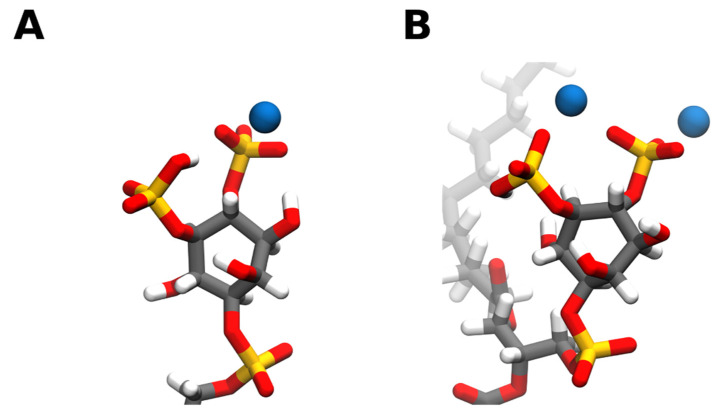
Snapshots of calcium ions interacting with the PI(4,5)P_2_ headgroup phosphates. Calcium can bind to PI(4,5)P_2_ either solely near the 4-phophomonoester (**A**) or in between the phosphomonoester groups (**B**). However, simultaneous binding between the two phosphomonoester groups is approximately 10 kcal/mol more unfavorable [31]. Carbon atoms are colored in grey, hydrogen in white, oxygen in red, phosphorus in orange, and calcium in blue. Snapshots obtained from a simulation of a bilayer consisting of 95:5 mol ratio POPC: PI(4,5)P_2_ in the presence of calcium in a 5:1 calcium to PI(4,5)P_2_ ratio, using the CHARMM36m forcefield run in GROMACS2019. Images were modeled using VMD.

Through hybrid Quantum mechanics/molecular mechanics (QM/MM) experiments we can get an insight on the molecular basis for cation binding to PI(4,5)P_2_ [31]. From a molecular point of view, when binding to a single PI(4,5)P_2_ lipid, both calcium and magnesium bind to PI(4,5)P_2_ either in between the phosphomonoester groups (Figure 4B) or solely near the 4-phophomonoester (Figure 4A). However, simultaneous binding between the two phosphomonoester groups is approximately 10 kcal/mol more unfavorable [31]. Divalent cation binding to the phosphodiester group has also been observed [67].

When analyzing the free energy associated with the removal of each divalent cation from its binding position, significantly more energy is required to remove calcium into the bulk water than it is for magnesium. The difference in free energy could come from the fact that, in contrast to calcium, magnesium appears to retain its first hydration shell in its equilibrium binding position. This causes its equilibrium binding position to be further away from the headgroup and leads to the formation of fewer hydrogen bonds, on average, between the headgroup and the surrounding water molecules. Interestingly, it was also shown that upon binding to calcium, the remaining PI(4,5)P_2_ headgroup proton at physiological pH could be favorably displaced and that the effective size of the PI(4,5)P_2_ headgroup would significantly decrease [31]. In the presence of magnesium, the dissociation of the remaining proton was not favorable, however, the decrease in effective headgroup surface area was also observed albeit to a lesser extent. All of these cation-induced changes can and will affect PI(4,5)P_2_ dynamics, thus influencing local membrane dynamics as well as its interactions with protein binding partners. 

**Figure 5:**
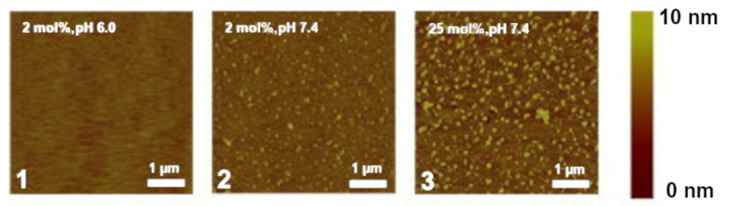
Snapshots of experiments on mixed lipid monolayers, containing different mol % of 1-stearoyl-2-oleoyl-*sn*-glycero-3-phosphocholine (SOPC) and PI(4,5)P2, while exposed to calcium. Reprinted from Biophysical Journal, 101, Ellenbroek, W.G.; Wang, Y.H.; Christian, D.A.; Discher, D.E.; Janmey, P.A.; Liu, A.J. Divalent cation-dependent formation of electrostatic PIP2 clusters in lipid monolayers. 2178–2184, Copyright (2011), with permission from Elsevier [69].

Apart from simply binding to PI(4,5)P_2_, both divalent cations also have the ability to crosslink PI(4,5)P_2_ lipids. This induces the formation of very stable cation-induced PI(4,5)P_2_ nanodomains. It has been shown through different experimental techniques that divalent cations, and especially calcium, are able to induce the formation of PI(4,5)P_2_ nanodomains, even at physiological concentrations of cation and lipid. In lipid monolayers, these clusters can be detected through AFM [68,69] (Figure 5). Through the use of fluorescent analogs of PI(4,5)P_2_ , calcium-induced clusters were shown to occur in model membranes at physiologically relevant concentrations of both calcium and PI(4,5)P_2_ [70]_._ Other phosphoinositides have also shown some propensity to form cation-induced clusters. PI(3,5)P_2_ has been found to form nanodomains by itself in the presence of physiological concentrations of calcium cations, however, in the presence of magnesium clustering was negligible [71]. On top of that, the clusters formed by PI(3,5)P_2_ were much smaller and likely less stable than those formed by PI(4,5)P_2_ [71]. On the other hand, when the monophosphorylated PI(4)P was tested in the same type of experiments, no calcium-induced clusters were observed [71]. 

**Figure 6:**
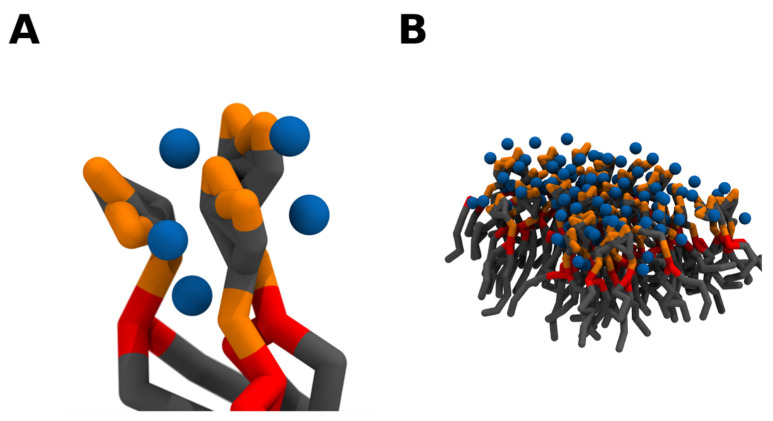
Crosslinking of PI(4,5)P_2_ lipids induces the formation of PI(4,5)P_2_ nanodomains. As a single divalent cation can bind up to 2 PI(4,5)P_2_ lipids and each lipid can potentially bind 3 divalent cations, a network of electrostatic interactions can crosslink PI(4,5)P_2_ lipids together (**A**). As the number of clustered lipids increases, PI(4,5)P_2_ nanodomains are formed (**B**). Coarse grain beads representing the inositol headgroup and acyl-chains are colored in grey, the glycerol component in red, the phosphate groups in orange, and calcium in blue. Snapshots obtained from a simulation of a bilayer consisting of 95:5 mol ratio POPC: PI(4,5)P_2_ in the presence of calcium in a 5:1 calcium to PI(4,5)P_2_ ratio, using the martini 2.2 coarse-grained forcefield run in GROMACS2019. Images were modeled using VMD.

From a molecular point of view, a single divalent cation can likely crosslink up to 2 PI(4,5)P_2_ lipids by simultaneously binding each lipid phosphodiester and/or headgroup phosphomonoester groups via strong electrostatic interactions [72]. A single PI(4,5)P_2_ lipid, however, can simultaneously bind up to 3 divalent cations, and thus be complexed with 3 other PI(4,5)P_2_ lipids (Figure 6). This net of PI(4,5)P_2_—cation interactions can thus induce the formation of a grid of tightly crosslinked lipids. Whilst the main driving force behind the clustering appears to be cation crosslinking, the formation of a complex network of intermolecular hydrogen bonds, between the headgroup hydroxyl and phosphomonoester groups, very likely plays a role in thermodynamically favoring clustering. Due to the electrostatic nature of the cation interactions, the propensity to crosslink PI(4,5)P_2_ lipids appears to be highly correlated with the affinity towards the divalent cation. Thus, Ca^2+^ shows a much greater clustering propensity than magnesium. In fact, Ca^2+^ induced clusters have been shown to be significantly larger than those induced by magnesium at the same experimental conditions [68]. However, although magnesium has a much weaker affinity for PI(4,5)P_2_ when compared to calcium, its steady-state levels are several orders of magnitude higher than those of calcium, and at these mM concentrations, it is also able to induce comparable PI(4,5)P_2_ clustering [71]. 

As the formation of these clusters is driven mainly by the crosslinking of the phosphate groups, the nanodomains formed are composed of almost only PI(4,5)P_2_. Studies have shown that other phosphorylated PI species can co-cluster with PI(4,5)P_2_, albeit to a lesser degree, but that the parent lipid phosphatidylinositol cannot [73]. Incorporation into clusters also seems to be independent from acyl-chain composition [73], however, it is very likely that different acyl-chain compositions induce the formation of nanodomains with different biophysical properties.

These cation-induced PI(4,5)P_2_ nanodomains are much more than simply the sum of their elements. While calcium is known to directly regulate the interaction of different protein domains to PI(4,5)P_2_ [74], the structure and dynamics of the phospholipid within the divalent cation-induced cluster present markedly distinct biophysical characteristics than the monodisperse lipid. As mentioned previously, binding of divalent cations, and in particular calcium, can alter PI(4,5)P_2_ headgroup exposure leading to a decrease in solvent-accessible area [31]. Additionally, as divalent cations accumulate, significant screening of the headgroup charges occurs, essentially shielding the large negatively charged headgroup from potential binding partners [75]. As PI(4,5)P_2_ lipids are forced to accumulate in an enclosed area, further reorganization of the headgroups occurs, promoted by the molecular interactions of the divalent cations with the 3 phosphate groups [76], effectively altering the headgroup conformation. This local accumulation likely influences PI(4,5)P_2_ acyl-chain dynamics and, therefore, local membrane order. Studies have shown that PI(4,5)P_2_ nanodomains have significantly reduced lateral dynamics [70] and that PI(4,5)P_2_, which as a single lipid has a strong preference for disordered domains, displays significantly less affinity for disordered domains upon clustering [71]. All of these altered biophysical properties can, and most likely will, influence downstream PI(4,5)P_2_ signaling by modulating its interactions with protein and lipid partners.

Altogether, these findings show that divalent cation-mediated clustering can lead to the formation of specific sites in the membrane highly enriched in PI(4,5)P_2_ while depleting the rest of the membrane [70]. PI(4,5)P_2_ is likely constitutively clustered in the membrane, crosslinked by Mg^2+^ ions alongside other minor phosphoinositide lipids. In the vicinity of active calcium channels, where calcium concentrations increase significantly upon opening of a channel, both ions will simultaneously contribute to the formation of the nanodomains, to form larger and more stable PI(4,5)P_2_ nanodomains. These cation-induced nanodomains can influence not only PI(4,5)P_2_ lateral organization and biophysical properties but also the way proteins interact with PI(4,5)P_2_, by modulating their localization in the plasma membrane, their target recognition and binding affinity to PI(4,5)P_2_, and even further downstream interactions with other proteins. Therefore, beyond the impact of calcium on PI(4,5)P_2_ levels in the membrane through activation of phospholipase activity, the direct interaction of divalent cations with PI(4,5)P_2_ is expected to play a crucial role in the regulation of the biological activity of this phospholipid.

### 4.3. Effect of Cholesterol on PI(4,5)P2 Properties and Distribution

Cholesterol is a crucial membrane component, implicated in a myriad of membrane processes. However, its most noted role is in the regulation of plasma membrane biophysical properties as a “fluidity buffer”. Whilst its effects can vary with different cholesterol contents, cholesterol, in general, decreases membrane fluidity by increasing lipid packing even leading to the cholesterol-dependent formation of coexisting liquid phases [77]. Like all the other phospholipids in the plasma membrane, PI(4,5)P_2_ is also subject to these cholesterol-dependent effects. 

**Figure 7:**
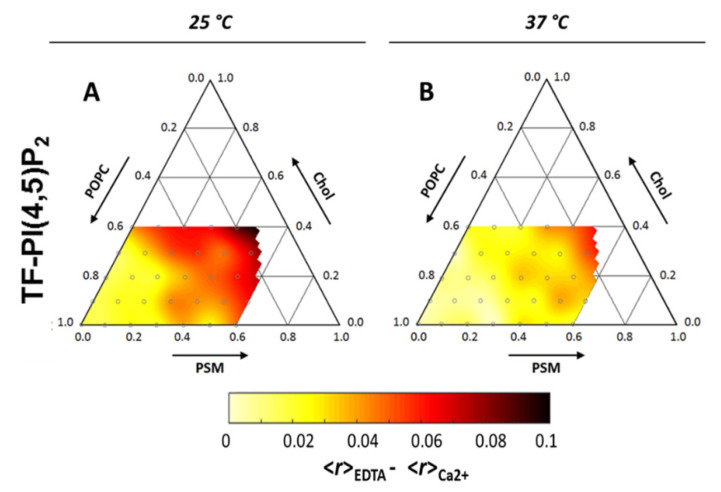
Ternary diagram for the POPC:PSM:Chol lipid mixture at 25 °C (**A**) and 37 °C (**B**). Color-code depicts decrease in measured fluorescence anisotropies of a PI(4,5)P_2_ fluorescent analog (TF-PI(4,5)P_2_) upon inclusion of 100 µM Ca^2+^. Since the decrease reflects homo-FRET between analogs incorporated within the same clusters, darker areas correspond to more efficient PI(4,5)P_2_ clustering. Adapted with permission from Sarmento, M.J.; Coutinho, A.; Fedorov, A.; Prieto, M.; Fernandes, F. Membrane order is a key regulator of divalent cation-induced clustering of PI(3,5)P_2_ and PI(4,5)P_2_. Langmuir 2017, 33, 12463–12477 [78]. Copyright (2017) American Chemical Society.

Unsurprisingly, given its large negatively charged headgroup and highly unsaturated acyl chain, PI(4,5)P_2_ was shown to preferentially partition into the less ordered cholesterol-poor phases of biphasic monolayers containing PI(4,5)P_2_:SOPC:Chol [78]. However, after the addition of calcium, the subsequent cation-induced PI(4,5)P_2_ nanodomains were shown to have increased the miscibility of the coexisting domains in the cholesterol-containing monolayers [78]. Related results have been observed, in a study with fluorescent derivatives of PI(4,5)P_2_ incorporated in ternary mixtures of POPC:SM:Chol. In this study, monodisperse PI(4,5)P_2_ presented low miscibility in more ordered lipid phases, yet, after cation-induced clustering, the preference for disordered domains decreased by more than two-fold [78]. Importantly, the lipid composition of this ternary mixture was shown to have a marked influence both on the extent of PI(4,5)P_2_ calcium-induced clustering and on the size of clusters formed (Figure 7) [78]. Since the dimensions of PI(4,5)P_2_ clusters were heavily dependent on temperature, it was concluded that the major factor regulating PI(4,5)P_2_ clustering was membrane order and not the presence of a specific molecular partner in the membrane. This suggests that the insertion of PI(4,5)P_2_ in more ordered domains is stabilized by the formation of cation-induced nanodomains. In a cellular context, the effect of cholesterol on PI(4,5)P_2_ appears to be heavily dependent on cell type. In fibroblasts [79] and cultured pancreatic β-cells [80], cholesterol depletion leads to decreased levels of free PI(4,5)P_2_, whilst in HEK293 [81] cholesterol enrichment was shown to promote PI(4,5)P_2_ depletion. 

Altogether, the findings we have previously discussed appear to be in agreement with theories that associate PI(4,5)P_2_ with the controversial cholesterol-rich microdomains, such as “lipid rafts” and caveolae, which are said to be involved in regulating a variety of membrane functions. PI(4,5)P_2_ has been found to be enriched in detergent-resistant membranes [82]. Moreover, while detergent extraction has been put into question on whether it induces an artefactual enrichment in PI(4,5)P_2_ [83], studies have shown PI(4,5)P_2_ to colocalize with more ordered membrane domains [84,85,86,87] and to be sensitive to membrane curvature [87,88], both associated with these types of structures. Interestingly, in a study where a PI(4,5)P_2_-specific phosphatase was targeted to either the “raft” or the “nonraft” membrane fractions of T cells, the authors were able to show clear evidence of compartmentalization of PI(4,5)P_2_-dependent signaling in each of the fractions. When depleting the “nonraft” fraction of PI(4,5)P_2_, T cells showed an increase in cell filopodia and cell spreading, whilst in contrast, when depleting the “raft” fraction of PI(4,5)P_2_, T cells showed smooth membranes free of ruffling and filopodia among other effects. Findings also appear to suggest that roughly half of the PI(4,5)P_2_ content is synthesized preferentially in these cholesterol-enriched domains [82]. Nonetheless, this is still an area of great controversy amongst researchers, and there is a lot yet to uncover before significant conclusions can be drawn on the importance of these microdomains for PI(4,5)P_2_ lateral organization. 

### 4.4. Effect of the Cytoskeleton and Curvature on PI(4,5)P2 Lateral Organization

PI(4,5)P_2_ has been shown to be a major player in cytoskeleton dynamics, by interacting and regulating the activity of numerous enzymes and cytoskeletal proteins [89,90]. However, the cytoskeleton can also regulate PI(4,5)P_2_, and in particular its lateral organization, via corralling by the cortical actin network. Cortical actin networks have been shown to be able to induce spatio-temporal confinement of phospholipids in the plasma membrane of living cells [91]. PI(4,5)P_2_ should be no exception to this effect and, in fact, due to its close proximity with a variety of actin-binding proteins [90], one can suspect it could be even more susceptible to these effects. Studies have shown that the cytoskeleton is responsible for some of the low mobility of PI(4,5)P_2_ in atrial myocytes [92]. 

Curvature can also greatly influence PI(4,5)P_2_ lateral organization. PI(4,5)P_2_ has been found to undergo a transient increase at the phagocytic cup during the initiation of phagocytosis [93,94]. More recently, it was found that the curvature induced by the engagement of non-biological solid particles with the plasma membrane was enough to increase PI(4,5)P_2_ concentrations at the site of contact. Additionally, as we previously discussed, PI(4,5)P_2_ has been associated with several stages of endocytosis and exocytosis, where curvature effects are paramount [40]. As a monodisperse lipid, PI(4,5)P_2_ has an inverted cone-shaped structure [95] due to its very large inositol headgroup. As such, it is associated with positive membrane curvature. After interacting with divalent cations, however, PI(4,5)P_2_ presents a cone-shaped structure [95], likely due to the decrease in headgroup area as well as the aggregation of the headgroups after complexation with the cations. In this case, it would be associated with negative membrane curvature. Whether local curvature at the plasma membrane plays a major role in dictating PI(4,5)P_2_ lateral organization or PI(4,5)P_2_ lateral organization contributes to local curvature is not entirely clear. In a cellular context, it is likely dependent on the process in question and the overall result of both effects. 

## 5. Concluding Remarks

Despite having been discussed separately in this review, all the complex biophysical properties we discussed previously are tightly interlinked processes. Furthermore, it is the combined effect of these properties that allows PI(4,5)P_2_ to be a major regulator of membrane dynamics despite being present at very low overall concentrations. While a lot of research has been conducted on these effects, many are still to be fully characterized, especially those associated with cation-induced nanodomains, such as the lipid conformation in these structures, the extent of charge dissipation, and the effect of these nanodomains on the local bilayer properties. A good molecular understanding of these effects is fundamental in order to better understand how PI(4,5)P_2_ carries out its role as a major plasma membrane regulator. 

Over the last decades, extensive research efforts have uncovered a multitude of different cellular roles of PI(4,5)P_2_. However, the current view on the majority of the mechanisms associated with these functions neglects the almost certain presence of a highly significant, if not dominant, pool of this phospholipid that is not monodispersed. Special consideration should be given to the fact that in the plasma membrane, PI(4,5)P_2_ must be either protein-bound or constitutively complexed with divalent cations within small clusters. PI(4,5)P_2_ within these structures is bound to have significantly different properties from the monodisperse lipid. These properties can influence PI(4,5)P_2_ interactions with its binding partners (such as proteins) as well as downstream protein-protein interactions. It is conceivable that many of PI(4,5)P_2_ cellular functions are also regulated by the extent of this effect.
